# Deep vein thrombosis prophylaxis in patients who undergo knee arthroscopy: a systematic review

**DOI:** 10.1186/s43019-024-00250-5

**Published:** 2024-12-05

**Authors:** Udit Dave, Emma G. Lewis, Victoria K. Ierulli, Shreya M. Saraf, Mary K. Mulcahey

**Affiliations:** 1 Tulane University School of Medicine, New Orleans, LA USA; 2 Department of General Surgery, Morristown Medical Center, Morristown, NJ USA; 3 Department of Orthopaedic Surgery and Rehabilitation, Loyola University Medical Center, 2160 S First Avenue, Maguire Building, Suite 1700, Maywood, IL 60153 USA

**Keywords:** Deep vein thrombosis, Venous thromboembolism, DVT prophylaxis, Factor Xa inhibitor, Aspirin, Knee arthroscopy

## Abstract

**Background:**

Knee arthroscopy is one of the most common procedures performed by orthopedic surgeons. A potentially life-threatening complication following this procedure is deep vein thrombosis (DVT). DVT prophylaxis can be obtained both mechanically (e.g., compression stockings) and chemically (e.g., aspirin, anticoagulants, and factor Xa inhibitors). Currently, there is no standardized guideline for DVT prophylaxis following knee arthroscopy. The purpose of this systematic review was to summarize how DVT prophylaxis is employed for patients who undergo knee arthroscopy.

**Methods:**

PubMed, Embase, and Cochrane Library were searched for studies published after 1998 according to Preferred Reporting Items for Systematic Reviews and Meta-Analyses (PRISMA) guidelines. Studies were included if they evaluated DVT prophylaxis regimens in patients of any age who underwent knee arthroscopy. Studies not written in English, that analyzed animals or cadavers, that did not directly evaluate patients undergoing knee arthroscopy, or that did not address DVT prophylaxis were excluded.

**Results:**

The initial search identified 300 studies, 15 of which were included. These 15 studies examined methods of DVT prophylaxis, including compression stockings (2 of 18; 11%), aspirin (1 of 18; 6%), factor Xa inhibitors (2 of 18; 11%), low-molecular-weight heparin (12 of 18; 67%), and neuromuscular electrical stimulation (1 of 18; 6%). Overall, 7 of 15 (47%) studies recommended DVT prophylaxis in all patients, and 3 (20%) studies supported its use for high-risk patients. Five (33%) studies did not support DVT prophylaxis, citing low incidence of postoperative DVT.

**Conclusions:**

Compression stockings, aspirin, factor Xa inhibitors, and low-molecular-weight heparin (LMWH) were identified as possible options for DVT prophylaxis in patients undergoing knee arthroscopy. For high-risk knee arthroscopy patients, factor Xa inhibitors and LMWH drugs are appropriate for DVT prophylaxis.

*Level of evidence* Level III, systematic review of level I–III studies.

## Background

Arthroscopic knee procedures account for a large portion of all procedures performed by orthopedic surgeons [1, 2]. Several potential complications can occur following knee arthroscopy, such as instability, anterior knee pain, decreased range of motion, infection, and deep venous thrombosis (DVT) [2–4]. Venous thromboembolism (VTE), which is a term that includes both DVT as well as pulmonary thromboembolism, is a life-threatening complication that can occur after knee arthroscopy. Despite the relatively low incidence compared with other more common postoperative complications associated with knee arthroscopy such as anterior knee pain, limited knee range of motion, bleeding, and infection, it is important to determine an effective methodology to prevent thromboembolic events following knee arthroscopy owing to their life-threatening potential [2].

Previous studies have suggested that DVT prophylaxis may be effective in reducing the risk of DVT. However, while standardized guidelines for VTE prevention exist for patients undergoing arthroplasty, no such guidelines are currently available for patients undergoing knee arthroscopy [5–7]. Furthermore, owing to variations in methodologies and DVT patient symptomatology in previous randomized controlled trials (RCTs), establishing an overarching guideline that assesses for the risk of DVT and effectiveness of prophylaxis is difficult [8]. Additionally, appropriately stratifying individual patients’ risk factors for DVT is important in determining reliable dosages that account for risk–benefit by successfully mitigating DVT incidence without bringing about a wide array of potentially dangerous side effects.

The purpose of this systematic review was to summarize how DVT prophylaxis is employed for patients who undergo knee arthroscopy. We hypothesized that LMWH and factor Xa inhibitors are effective DVT prophylaxis methods with a safe side-effect profile.

## Methods

### Literature search methodology

A comprehensive search of PubMed, Embase, and Cochrane Library databases was performed in accordance with the Preferred Reporting Items for Systematic Reviews and Meta-Analyses (PRISMA) guidelines. The search was performed in March 2023, and the full search strategy utilized can be found in Appendix 1.

Studies were included if they evaluated male and female participants of any age group who underwent knee arthroscopy, were prospective randomized controlled trials or retrospective cohort studies, evaluated DVT prophylaxis regimens in patients undergoing knee arthroscopy, in the English language, and were published from 1998 to 2022. Studies that were cadaveric or translational, did not directly evaluate patients undergoing knee arthroscopy, did not study DVT prophylaxis, or had study designs such as systematic reviews, observational studies, conference abstracts or case reports were excluded. The search was performed by two authors (U.D. and E.G.L.) using the online software program Covidence (Veritas Health Innovation Ltd, Melbourne, Australia) to independently screen titles, abstracts, and full article texts. Any disagreements were resolved through discussion between these two authors and consensus with a third senior author (M.K.M.).

### Data extraction and quality assessment

Data items extracted from each study included the type of DVT prophylaxis used, comparisons of risk factors for DVT incidence in patients undergoing arthroscopic knee procedures, and measurements of DVT prophylaxis effectiveness. Data quality assessment for randomized controlled trials was performed using the revised version 2 of the Cochrane risk-of-bias tool for randomized trials (RoB 2) [9]. Data quality assessment for nonrandomized prospective studies and retrospective studies was performed with the methodological index for nonrandomized studies (MINORS) criteria [10]. An attempt to prevent potential biases or misidentification in included texts was accomplished by disclosing which studies presented data in addition to information about the effectiveness of DVT prophylaxis. Significant heterogeneity exists between the studies that were ultimately included for data extraction with regards to surgical context (i.e., ligamentous injuries versus no ligamentous injuries), DVT prophylaxis method used, and level of evidence (i.e., RCTs versus prospective non-randomized studies versus retrospective studies). Owing to the numerous variations across these categories, we did not perform formal quantitative heterogeneity analysis with the calculation of *I*-squared values as the grouping of any of these studies by one given category would be arbitrary and introduce substantial bias. Furthermore, as a result of this high heterogeneity, we were unable to justify the pooling of data from this systematic review into a meta-analysis and instead opted to perform a qualitative data comparison.

## Results

### Study characteristics and study quality/risk of bias

**Fig. 1:**
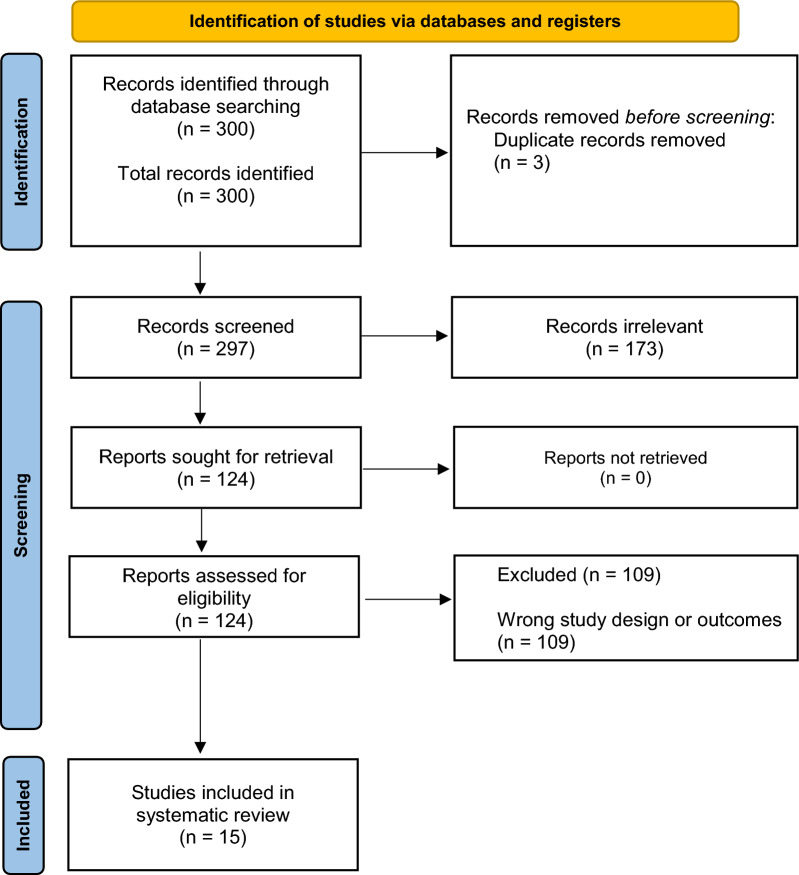
Preferred Reporting Items for Systematic Reviews and Meta-Analyses (PRISMA) study selection flow diagram. The numbers of screened, excluded, and included studies are shown

A total of 300 studies were identified in the initial search, 3 of which were duplicates and were subsequently excluded. The remaining 297 studies underwent title and abstract screening; 173 were found to be irrelevant to the study aims and therefore excluded. The remaining 124 studies were assessed for eligibility with full-text review. After excluding 109 studies for having an incorrect study design or outcomes, 15 studies were included for data extraction (Fig. 1).

**Fig. 2:**
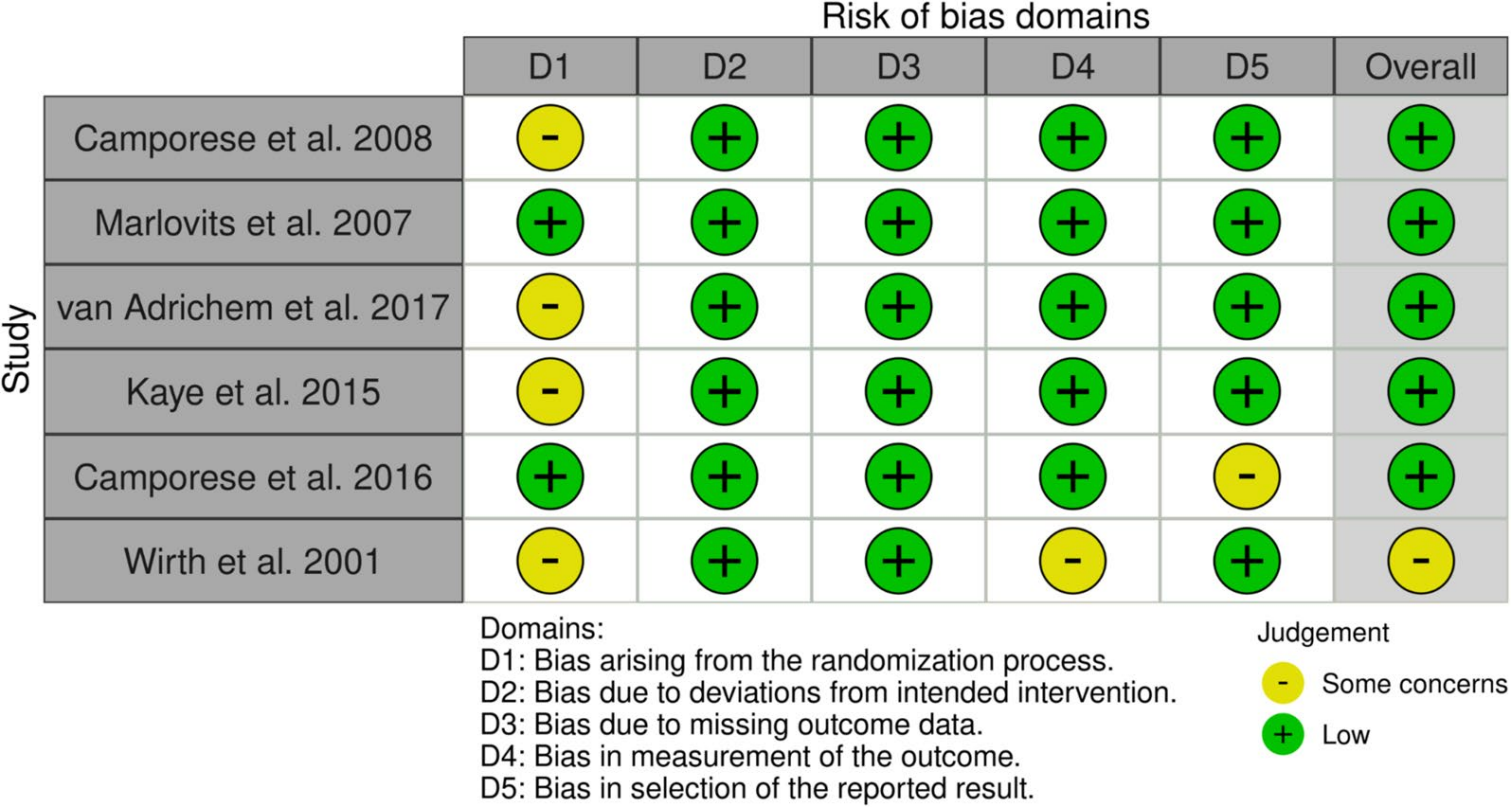
Summary of study quality and risk of bias assessment based on version 2 of the Cochrane risk-of-bias tool for randomized trials (RoB 2)

Table 1 summarizes study quality based on methodological index for nonrandomized studies (MINORS) criteria for nonrandomized studies [10]. The ideal MINORS score for noncomparative studies is 16, with scores ≤ 8 being the accepted cutoff for poor study quality. Each of the included studies had a score ≥ 10, indicating sufficiently low risk of bias and moderate to high study quality. Figure 2 summarizes study quality based on the Cochrane RoB 2 tool for RCTs [9]. Each of the studies included in this systematic review and meta-analysis was an RCT with level 1 evidence. The RoB 2 tool divides bias into five different domains: the randomization process, deviations from intended interventions, missing outcome data, outcome measurement, and selection of reported results [9]. Determination of bias in each domain is then used to produce an overall risk of bias determination for the RCT, ranging from “low risk of bias,” to “some concerns,” to “high risk of bias.” Each RCT included in this systematic review had sufficiently low risk of bias for inclusion.

**Table 1:** Summary of study quality and risk of bias assessment based on methodological index for nonrandomized studies (MINORS) criteria

Study (year)	A clearly stated aim	Inclusion of consecutive patients	Prospective collection of data	Endpoints appropriate to the aim of the study	Unbiased assessment of the study endpoint	Follow-up period appropriate to the aim of the study	Loss to follow-up less than 5%	Prospective calculation of the study size	Total
Jetty et al. [10]	2	2	0	2	0	2	2	0	10
Hoppener et al. [14]	2	2	2	2	0	2	2	0	12
Yeo et al. [17]	2	2	2	2	0	2	2	0	12
Schippinger et al. [18]	2	2	2	2	0	2	2	0	12
Xiong et al. [19]	2	2	0	2	0	2	2	0	10
Muñoa et al. [20]	2	2	0	2	0	2	2	0	10
Chen et al. [21]	2	2	0	2	0	2	2	0	10
Adala et al. [22]	2	2	2	2	0	2	2	0	12
Dong et al. [23]	2	2	2	2	0	2	2	0	10

A total of 15 studies were included in this systematic review. Table 2 summarizes study characteristics. Studies that were included analyzed a variety of DVT prophylaxis methods. Jetty et al. conducted a retrospective analysis of 10 patients with known familial thrombophilia and compared them with 110 normal subjects who underwent knee arthroscopy to evaluate a potential relationship between postoperative DVT and familial thrombophilia [11]. The authors did not specify the types of arthroscopic knee procedures included in their analysis. Compared with the hematologically normal subject group, subjects with high homocysteine (three patients, *p* = 0.02), factor V Leiden heterozygosity (four patients, *p* = 0.0004), and high factor VIII level (five patients, *p* = 0.0011) all had a higher incidence of DVT, which was statistically significant. A total of 21 patients developed a DVT, 8 (40%) of whom had a familial thrombophilia [11].

**Table 2:** Characteristics of studies included in the systematic review

Authors	Year	Study type	Number of patients	Types of procedures included	DVT prophylaxis methods
Jetty et al. [11]	2016	Retrospective	120	Knee arthroscopy (not specified)	None
Camporese et al. [12]	2008	RCT	1317	Knee arthroscopy including ligament reconstruction	LMWH and compression stockings
Marlovits et al. [13]	2007	RCT	175	Only ACL reconstruction	LMWH
van Adrichem et al. [14]	2017	RCT	1543	Knee arthroscopy without ligament reconstruction	LMWH
Hoppener et al. [15]	2006	Prospective nonrandomized	335	Knee arthroscopy without ligament reconstruction	None
Kaye et al. [16]	2015	RCT	170	Knee arthroscopy including ligament reconstruction	Aspirin
Camporese et al. [17]	2016	RCT	234	Knee arthroscopy including ligament reconstruction	Rivaroxaban
Yeo et al. [18]	2016	Prospective nonrandomized	1410	Knee arthroscopy (not specified)	None
Schippinger et al. [19]	1998	Prospective nonrandomized	101	Knee arthroscopy without ligament reconstruction	LMWH
Xiong et al. [20]	2022	Retrospective	278	Only ACL reconstruction	Neuromuscular electrical stimulation (NMES)
Muñoa et al. [21]	2014	Retrospective	467	Knee arthroscopy including ligament reconstruction	Rivaroxaban and bemiparin
Chen et al. [22]	2017	Retrospective	128	Only PCL reconstruction	None
Adala et al. [23]	2011	Prospective nonrandomized	112	Only ACL reconstruction	None
Dong et al. [24]	2015	Prospective non-randomized	282	Only ligament reconstruction	None
Wirth et al. [25]	2001	RCT	239	Knee arthroscopy including ligament reconstruction	LMWH

DVT, deep vein thrombosis; RCT, randomized controlled trial; LMWH, low-molecular-weight heparin; ACL, anterior cruciate ligament; NMES, neuromuscular electrical stimulation; PCL, posterior cruciate ligament

Camporese et al. conducted a prospective study in 2008 that examined whether LMWH (*n* = 657) or compression stockings (*n* = 660) was more likely to reduce DVT incidence following arthroscopic knee procedures including partial meniscectomy, cartilage shaving, cruciate ligament reconstruction, synovial resection, and combinations of these procedures [12]. The authors found that 21 (3.2%) patients who wore compression stockings developed a DVT, as compared with only 10 (1.5%) patients in the LMWH cohort (*p* = 0.005). Additionally, they found that, out of all included procedures, only partial meniscectomies were independently associated with the development of DVT or all-cause mortality including bleeding events [12].

In 2007, Marlovits et al. conducted a prospective cohort study of 175 patients comparing DVT incidence in patients undergoing anterior cruciate ligament (ACL) reconstruction [13]. These patients received LMWH thromboprophylaxis either postoperatively in the hospital for a 20-day course after discharge. The authors sought to determine the effectiveness of long-term (20 days) DVT prophylaxis with LMWH versus only immediate postoperative prophylaxis. All 175 patients received 40 mg of subcutaneous enoxaparin immediately postoperatively. Patients were then subsequently divided into long-term LMWH versus placebo groups. The placebo group participants received self-administered subcutaneous placebo injections daily for 20 days, whereas the treatment group participants received self-administered subcutaneous 40 mg enoxaparin injections daily for 20 days. The authors found that the outpatient LMWH (*n* = 72) group had two confirmed DVTs (2.8%), while the placebo group (*n* = 68) had 28 (41.2%) (*p* < 0.001). No patients in either group developed a pulmonary embolism (PE) [13].

A 2017 prospective study by van Adrichem et al. assigned 1543 patients to receive either a prophylactic dose of LMWH for 8 days after undergoing knee arthroscopy (773 patients) or no anticoagulant therapy (770 patients) [14]. Venous thromboembolism occurred in 5 of 731 patients (0.7%) in the treatment group and 3 of 720 patients (0.4%) in the control group with a 95% confidence interval of mean difference of −0.6 to 1.2. The difference in means was not significant [14].

Hoppener et al. prospectively evaluated the risk of developing a DVT in 335 patients following arthroscopic knee procedures including meniscectomy, debridement, synovectomy, and loose body removal [15]. No patients undergoing ligament reconstruction were included in this study. None of the 335 patients were given any type of chemical or mechanical thromboprophylaxis. Each of the patients underwent a complete bilateral extended ultrasound (US) during their 2-week follow-up visit and 19 (5.7%) were found to have a DVT. In total, 11 (57.9%) of these patients had a partial meniscectomy, 5 (26.3%) had a debridement, 3 (15.8%) had a loose body removal, and 1 (5.3%) had a lavage. Of the 19 patients who developed a DVT, 2 (0.6%) were symptomatic and 1 (0.3%) experienced a nonfatal pulmonary embolism. The authors concluded that, even in the absence of DVT prophylaxis, rates of DVT incidence were sufficiently low and no specific high-risk groups could be identified [15].

Another prospective RCT evaluating DVT prophylaxis was performed by Kaye et al. with the primary goal of establishing the efficacy of aspirin as a postoperative venous thrombosis prophylaxis agent in patients undergoing knee arthroscopy [16]. Procedures performed in this study included meniscectomy, meniscus repair, chondroplasty, and ACL reconstruction. One hundred seventy patients were randomized to receive either 325 mg of oral aspirin daily for 14 days postoperatively (66 patients) or to the control group, which received no medication (104 patients). Compression venous duplex US was performed on bilateral lower extremities 10–14 days postoperatively to document the incidence of DVT. No cases of DVT were identified in either group [16].

Camporese et al. performed a prospective study in 2016 that examined rivaroxaban’s effect on the incidence of DVT compared with placebo in a total of 234 patients following arthroscopic knee procedures including ligament reconstruction [17]. Patients received 10 mg rivaroxaban once daily over the course of seven days postoperatively. They were then examined with ultrasound at postoperative day 7 or day 8, if they demonstrated any symptoms of DVT (i.e., tenderness, erythema). One of 120 patients in the rivaroxaban group developed a DVT compared with 7 of 114 patients in the placebo group (0.8% versus 6.1% respectively, *p* = 0.03). Differences in DVT incidence were not stratified by procedure type. No patients experienced any major bleeds, which can be a complication of anticoagulation therapy. Additionally, no association was found between the type of arthroscopic procedure and thrombotic events [17].

Yeo et al. conducted a prospective cohort study of 1410 patients to examine the incidence of DVT in patients undergoing knee arthroscopy who did not receive DVT prophylaxis [18]. The authors did not specify which types of procedures were included in their study. Patients were given mechanical prophylaxis and encouraged to ambulate on postoperative day 1, after which point, they were monitored for symptoms such as lower limb pain or swelling that are suggestive of a DVT. The incidence of DVT in this population was approximately 7 out of 1,410 (0.5%) and was found to be statistically insignificant [18].

Schippinger et al. performed a prospective cohort study to determine the incidence of post-knee-arthroscopy thromboembolic events in patients who took chemoprophylaxis [19]. Conditions treated in this study included meniscal tears, chondromalacia, arthrosis, medial shelf syndrome, and fat pad hypertrophy. No ligament reconstruction patients were included, and results were not stratified based on type of procedure performed. All 101 patients received 5000 IU of intramuscular LMWH at least 12 h preoperatively. Prior to surgery, patients were screened for common risk factors for DVT (i.e., obesity, varicose veins, contraceptive pills, and tobacco use). All patients were screened for DVT 5 weeks postoperatively via ultrasound. For patients with questionable ultrasound findings, phlebography was performed to determine DVT status. Overall, 12 of 101 (11.9%) patients had a thromboembolic event, 8 of 101 (7.9%) had a DVT, of which 4 (4.0%) had symptoms. Additionally, 9 of 101 (8.9%) patients developed a pulmonary embolism, of which 8 (7.9%) had symptoms. Finally, 5 (4.9%) of the patients who developed pulmonary embolism also had a DVT [19].

In 2022, Xiong et al. evaluated the clinical effects of neuromuscular electrical stimulation (NMES) as DVT prophylaxis for 278 patients undergoing ACL reconstruction [20]. A total of 154 (55.4%) patients received NMES in comparison with a control group of 124 (44.6%) patients that did not receive any DVT prophylaxis. Diameter and DVT color Doppler US screening results were performed on postoperative day 4 and revealed a statistically significant (*p* < 0.05) reduction in DVT incidence in NMES recipients compared with the control cohort [20].

Muñoa et al. conducted a retrospective analysis with 467 patients undergoing knee arthroscopy, comparing daily rivaroxaban 10 mg for three weeks beginning 6–8 h postoperatively (237 patients) versus bemiparin 3500 IU subcutaneously 24 h postoperatively (230 patients) [21]. Procedures included meniscectomy, ACL reconstruction, and complex microfracture repair with osteochondral allograft [21]. Patients were evaluated for symptoms of DVT at their one and 3-month follow-up visits. Data were not stratified based on the type of arthroscopic procedure performed. No thromboembolic events occurred in either group, and there were no statistically significant differences in outcomes between patients who received rivaroxaban versus those who received bemiparin [21].

Chen et al. conducted a retrospective cohort study to identify the incidence as well as associated risk factors for DVT following arthroscopic posterior cruciate ligament (PCL) reconstruction [22]. A total of 128 patients who underwent PCL reconstruction were retrospectively divided into two groups based on whether they developed a DVT postoperatively. Of the 128 patients, 28 (21.9%) developed a DVT. The authors identified older age, high D-dimer levels, and longer procedures requiring tourniquet use as substantial risk factors for developing a DVT following PCL reconstruction [22].

Adala et al. performed a prospective analysis to evaluate the incidence of DVT in 112 patients (61 male and 51 female) undergoing arthroscopic ACL reconstruction [23]. All patients received a Doppler ultrasound venous scan 1 day preoperatively, day 3 postoperatively (when they were discharged), and at the 4 week follow-up visit. No patients received any form of DVT prophylaxis for the duration of the study. In total, two male patients (1.8%) developed DVTs, which were confirmed by Doppler venous scan. The authors found that the incidence of DVT in patients undergoing arthroscopic ACL reconstruction was 1.8% [23].

Dong et al. prospectively investigated the incidence of DVT in 282 Chinese patients undergoing ligament reconstruction involving the ACL, medial collateral ligament (MCL), lateral collateral ligament (LCL), and PCL [24]. Patients undergoing revision procedures were included. DVT was present in 34 of 282 patients (12.1%), and clinical diagnoses were confirmed via color Doppler ultrasound. Overall, 11 (7.2%) DVTs occurred in a total of 152 patients who underwent ACL reconstruction [24].

Wirth et al. performed a RCT to compare the incidence of DVT following elective knee arthroscopy in patients who were and were not receiving LMWH anticoagulation therapy [25]. A total of 239 patients were evaluated, 122 (51%) did not receive DVT prophylaxis and 117 (49%) received LMWH once daily subcutaneously for 7–10 days postoperatively. A total of five DVTs (4.1%) were detected in the control group and one in the LMWH group (0.85%). No patients were reported to be symptomatic. The relative risk reduction of reviparin prophylaxis was estimated to be 80%. These results were not further stratified by the type of procedure performed [25].

Overall, seven studies supported the use of DVT prophylaxis in all patients undergoing knee arthroscopy for any reason, three studies supported it for high-risk patients, and five studies did not support the use of DVT prophylaxis in any patients undergoing knee arthroscopy. However, it is important to consider the significant heterogeneity between studies with regards to study design, DVT prophylaxis method employed, type of operation (i.e., with or without ligamentous reconstruction), and patient population.

## Discussion

This study demonstrated that recommendations for DVT prophylaxis in patients undergoing knee arthroscopy vary. Overall, 7 of 15 (47%) studies advocate for some type of DVT prophylaxis in all patients undergoing knee arthroscopy through methods such as low-molecular-weight heparin, factor Xa inhibitors, and neuromuscular electrical stimulation. In total, 3 of 15 (20%) studies advocated for DVT prophylaxis in high-risk patient populations (e.g., patients with thrombophilia and Asian populations). While 5 of 15 (33%) studies suggested that DVT prophylaxis is not necessary for patients undergoing knee arthroscopy.

Overall, 7 (47%) studies concluded that DVT prophylaxis had clear benefits in patients undergoing knee arthroscopy. In the 2008 study by Camporese et al., the authors found that prophylactic LMWH effectively reduced the incidence of venous thrombosis and it was more effective than compression stockings [12]. Additionally, Marlovits et al. concluded that extended duration outpatient LMWH significantly reduced the incidence of DVT in patients undergoing ACL reconstruction compared with short-term inpatient LMWH therapy [13]. In their 2016 study, Camporese and colleagues determined that a one week course of rivaroxaban (a factor Xa inhibitor) 10 mg can be safely utilized for thromboprophylaxis after knee arthroscopy [17]. Similar findings were reported by Schippinger et al., who concluded that all patients require routine use of thromboprophylaxis and that the appropriate dose for this prophylaxis is likely higher than the dose typically given [19]. Xiong et al. used mechanical rather than chemical DVT prophylaxis and found that neuromuscular electrical stimulation can effectively reduce pain, knee swelling, and incidence of DVT in patients following ACL reconstruction [20]. However, Muñoa et al. concluded that extended prophylaxis with rivaroxaban 10 mg for 3 weeks is equally as effective as bemiparin (a low-molecular-weight heparin) following knee arthroscopy [21]. A need for chemical prophylaxis was also outlined by Wirth et al., who found that patients undergoing knee arthroscopy had a moderate risk of thromboembolic events following surgery and that LMWH can provide effective prophylaxis [25]. It is important to note that six of the seven studies that advocated for DVT prophylaxis in patients undergoing knee arthroscopy included patients who underwent ligamentous reconstruction and two exclusively evaluated DVT prophylaxis in the context of ACL reconstruction. Given the increase in DVT risk associated with more invasive procedures such as ligamentous reconstruction, it is likely that these surgical factors influenced the outcomes of the studies and the conclusion of the authors to support DVT prophylaxis for knee arthroscopy. Previous literature has reported the incidence of DVT following knee arthroscopy to be 10%, and that chemoprophylaxis with LMWH or a factor Xa inhibitor, such as rivaroxaban, is warranted given the risks and benefits of anticoagulating [2, 7, 26]. A large proportion of studies included in our systematic review (47%) strongly advocate for some form of DVT prophylaxis, and this recommendation is validated by the high rate of DVT reported by previous literature [2, 7, 26].

In total, three studies concluded that DVT prophylaxis in high-thromboembolism risk patients undergoing knee arthroscopy had protective benefits. Jetty et al. reported that it would be beneficial to incorporate preoperative prophylaxis protocols for high-risk patients with familial thrombophilia prior to undergoing arthroscopic knee procedures [11]. Chen et al. concluded the treatment of DVT with anticoagulants such as LMWH and batroxobin (snake-venom-derived anticoagulant) is effective after specific risk-factors for DVTs following knee arthroscopy are identified [27]. Similarly, Dong et al. found that DVT prophylaxis should be used for Chinese patients after undergoing knee arthroscopy to decrease the incidence of DVT [24]. Significant heterogeneity existed between these studies with regards to included procedures as Dong et al. only evaluated patients who underwent ligament reconstruction whereas Chen et al. more specifically evaluated exclusively patients who underwent PCL reconstruction and Jetty et al. did not specify which arthroscopic procedures their patients underwent.

A 2019 systematic review and meta-analysis by Huang et al. with 4290 knee arthroscopy patients demonstrated that DVT prophylaxis with anticoagulant therapy did not reduce DVT incidence with a rate ratio of 0.98 (95% CI 0.44–2.19; *I*^2^ value = 0%; *p* = 0.97) [28]. Furthermore, previous studies have concluded that for elective outpatient orthopedic procedures such as knee arthroscopy, postoperative DVT prophylaxis is not necessary owing to the relatively low associated rates of DVT [29–32]. This systematic review advocated against routine anticoagulation following knee arthroscopy, but conceded that risks for DVT incidence are constantly fluctuating, making it difficult to provide a conclusive “yes or no” on the need for DVT prophylaxis in patients undergoing knee arthroscopy. This suggests that patients who are at high-risk for developing a DVT (e.g., major thrombophilias) should be considered on an individual basis to determine whether DVT prophylaxis is necessary. Additionally, side effects of DVT prophylaxis, especially LMWH drugs, include increased bleeding risk and risk for secondary infections postoperatively, which must be weighed against a patient’s specific risk for developing a DVT in the setting of knee arthroscopy.

In total, five studies concluded that DVT prophylaxis was not required for patients undergoing knee arthroscopy, as it did not seem to provide a significant benefit. The 2017 study by van Adrichem et al. found that prophylaxis with LMWH for 8 days following knee arthroscopy versus DVT prophylaxis for the full period of immobilization due to casting was not effective for thromboembolism prophylaxis [14]. Similarly, Hoppener et al. did not recommended thrombophylaxis as a standard of care in patients undergoing knee arthroscopy because the authors did not find a connection between anticoagulation and prevention of developing a DVT [15]. Kaye et al. concluded that aspirin is not warranted for DVT prophylaxis following knee arthroscopy because it did not significantly reduce the incidence of DVT [16]. Yeo et al. concluded that, since the rates of DVT patients who were not put on DVT prophylaxis after knee arthroscopy were low, a protocol for DVT prevention in this population is unnecessary [18]. Finally, Adala et al. did not recommend routine thromboprophylaxis in patients undergoing ACL reconstruction owing to a low incidence of DVT postoperatively [23]. It is important to note that three of these studies included ligament reconstruction patients, one did not include ligament reconstruction patients, and one did not specify the type of arthroscopic procedures their patients underwent. A 2014 systematic review by Sun et al. with 3998 total eligible patients found a pooled risk ratio for the development of DVT to be 0.18 for those who used LMWH as DVT prophylaxis with an absolute risk reduction of 1.2% (1.5% reduced to 0.3% with DVT prophylaxis) [6]. The authors suggested that the incidence of DVT in patients undergoing knee arthroscopy was not significant enough to warrant every patient receiving prophylactic therapy [6].

Our study demonstrated that a large proportion of RCTs utilizing primary data advocated for DVT prophylaxis in all (7 of 15) or a high-risk subset (3 of 15) of patients. Only 5 of 15 studies suggested that no DVT prophylaxis was needed after knee arthroscopy, and many of the studies cited a low incidence rate of DVT following these types of procedures as the predominant driving force in decision making.

### Limitations

There are several limitations to this study. First, we limited our search to studies written in English. This may have excluded related studies written in other languages. Additionally, we were unable to stratify the results with more specific high-risk factors such as obesity, contraceptive pills, and tobacco use. Each of these factors would likely affect the safety and efficacy of various DVT prophylaxis measures. The inherent heterogeneity of our data given the multitude of DVT prophylaxis methods employed by the included studies also limits the conclusions that could be made. In particular, we included five studies that evaluated arthroscopic procedures including ligament reconstruction, five studies that only evaluated ligament reconstruction, three studies that excluded ligament reconstruction from their arthroscopic procedures, and two studies that did not specify which arthroscopic knee procedures were included. There was further variation in the types of DVT prophylaxis methods employed by each of these studies, and there was a lack of consensus between included studies regarding DVT prophylaxis guidelines. Furthermore, only 15 studies met inclusion criteria out of the 300 studies that were identified in our initial search, which may suggest that the findings of this systematic review have limited generalizability. Our study also has several strengths. We were able to conduct a comprehensive search strategy with overlapping approaches and discussion to review inclusion and exclusion criteria. We were also able to effectively include data that was specific to knee arthroscopy and DVT prophylaxis intervention selection.

Further studies directly comparing various DVT prophylaxis methods utilized following knee arthroscopy could help clarify the relative effectiveness and the risk–benefit of side-effect profiles for each medication.

## Conclusions

Compression stockings, aspirin, factor Xa inhibitors, and low-molecular-weight heparin (LMWH) were identified as possible options for DVT prophylaxis in patients undergoing knee arthroscopy. For high-risk knee arthroscopy patients, factor Xa inhibitors and LMWH drugs are appropriate for DVT prophylaxis.

## Data Availability

Data sharing is not applicable to this article as no datasets were generated or analyzed during the current study. The search terms used to conduct the systematic literature search have been included in the manuscript.

## Full search strategy

('dvt (deep vein thrombosis)'/exp OR 'dvt (deep vein thrombosis)' OR 'acute dvt'/exp OR 'acute dvt' OR 'acute deep venous thrombosis'/exp OR 'acute deep venous thrombosis' OR 'deep thrombo-phlebitis'/exp OR 'deep thrombo-phlebitis' OR 'deep thrombophlebitis'/exp OR 'deep thrombophlebitis' OR 'deep vein blood clots'/exp OR 'deep vein blood clots' OR 'deep vein thrombophlebitis'/exp OR 'deep vein thrombophlebitis' OR 'deep vein thrombosis'/exp OR 'deep vein thrombosis' OR 'deep vein thrombus'/exp OR 'deep vein thrombus' OR 'deep venous thrombophlebitis'/exp OR 'deep venous thrombophlebitis' OR 'deep venous thrombosis'/exp OR 'deep venous thrombosis' OR 'deep venous thrombus'/exp OR 'deep venous thrombus' OR 'recurrent dvt'/exp OR 'recurrent dvt' OR 'thrombosis, acute deep venous'/exp OR 'thrombosis, acute deep venous' OR 'cerebral embolism and thrombosis'/exp OR 'cerebral embolism and thrombosis' OR 'embolism and thrombosis'/exp OR 'embolism and thrombosis' OR 'embolism, thrombo'/exp OR 'embolism, thrombo' OR 'intracranial embolism and thrombosis'/exp OR 'intracranial embolism and thrombosis' OR 'thrombo embolic disease'/exp OR 'thrombo embolic disease' OR 'thrombo embolism'/exp OR 'thrombo embolism' OR 'thrombo-emboli'/exp OR 'thrombo-emboli' OR 'thrombo-embolus'/exp OR 'thrombo-embolus' OR 'thromboemboli'/exp OR 'thromboemboli' OR 'thromboembolic'/exp OR 'thromboembolic' OR 'thromboembolic complication'/exp OR 'thromboembolic complication' OR 'thromboembolic disease'/exp OR 'thromboembolic disease' OR 'thromboembolic process'/exp OR 'thromboembolic process' OR 'thromboembolism'/exp OR 'thromboembolism' OR 'thromboembolus'/exp OR 'thromboembolus' OR 'thromboemboly'/exp OR 'thromboemboly') AND ('patellofemoral instability'/exp OR 'patellofemoral instability' OR mpfl OR 'medial patellofemoral ligament reconstruction'/exp OR 'medial patellofemoral ligament reconstruction' OR 'medial patellofemoral ligament repair'/exp OR 'medial patellofemoral ligament repair' OR 'patellofemoral ligament'/exp OR 'patellofemoral ligament' OR (('patell* instability' OR 'patell* instability':ab,ti OR mpfl OR mpfl:ab,ti OR 'medial patellofemoral ligament*' OR 'medial patellofemoral ligament*':ab,ti) AND ('knee'/exp OR 'knee' OR knee*:ab,ti OR 'knee joint'/exp OR 'knee joint' OR femorotibia*:ab,ti) AND ('reconstruction'/exp OR reconstruction OR reconstruction*:ab,ti OR 'repair'/exp OR repair OR repair*:ab,ti OR 'surgery'/exp OR 'surgery' OR surger*:ab,ti))).

## Abbreviations

DVT: Deep vein thrombosis
LMWH: Low molecular weight heparin
RCT: Randomized controlled trial
PRISMA: Preferred Reporting Items for Systematic Reviews and Meta-Analyses
RoB 2: Version 2 of the Cochrane risk-of-bias tool for randomized trials
MINORS: Methodological index for nonrandomized studies
ACL: Anterior cruciate ligament
PCL: Posterior cruciate ligament
PE: Pulmonary embolism
US: Ultrasound
NMES: Neuromuscular electrical stimulation
MCL: Medial collateral ligament
LCL: Lateral collateral ligament
